# Plants Tell Caterpillars When It's Safe to Forage

**DOI:** 10.1371/journal.pbio.0040194

**Published:** 2006-05-16

**Authors:** Liza Gross

Light and temperature exert significant control over the daily lives of plants and animals. Many nocturnal species feed during the cover of night to escape a bevy of potential predators that hunt during the day. The effect of photoperiod (the 24-hour daylight cycle) is so great that zoos can trick nocturnal animals into becoming active for visitors simply by keeping their exhibit dark in the daytime and illuminating it at night.

But the world is filled with cues that could influence the daily feeding patterns of an organism. Many plants, for example, respond to foraging damage by releasing specialized chemical signals—volatile organic compounds that evaporate in the air—that attract the forager's natural enemies. This strategy is obviously no use against a cow, but proves effective when the offender is a caterpillar and the summoned predator is a wasp. Just how much control such biotic factors exert over a forager's daily routine has remained an open question. But in a new study, Kaori Shiojiri, Rika Ozawa, and Junji Takabayashi show that plant signals can indeed regulate herbivore behavior.

When the larvae of beet armyworms *(Spodoptera exigua)* feed on corn, the plant releases volatile compounds that act as a magnet for parasitic wasps *(Cotesia marginiventris)*, which deposit their eggs in the larvae. Production of volatile chemicals increases during the day (when wasps are active) and decreases at night, suggesting that variations in production might affect the daily activity patterns of foraging larvae, with low production sending the signal that the coast is clear.

To test this hypothesis, Shiojiri et al. exposed larvae of a corn-munching caterpillar, *Mythimna separata*, to volatile compounds from corn and varied the light and dark conditions for both corn and insect. Corn infested with *M. separata* releases volatiles that attract parasitic wasps *(C. kariyai)*. Previous work had established that *M. separata* is nocturnal, based on experimental observations of the larvae's daily feeding and hiding behavior on corn plants, but the impact of variable volatile production had not been examined.

In this study, the researchers separated the effects of photoperiod from that of host plant volatiles to tease out their relative contributions to caterpillar behavior. First, they tested the effects of light. If larvae are diurnal, they should hide in “shelters” fashioned out of filter paper attached to the plastic cups they were kept in. When larvae were fed an artificial diet, however, different light conditions produced no changes in their hiding behavior.

But introducing plants changed larvae behavior under both day and night conditions. Six pots of three uninfested corn plants—plants that had never been grazed—were placed around the cups of larvae. After eight hours, about 20% more larvae went into hiding when the lights were on and plants were added. And when plants were introduced under dark conditions, about 30% *less* larvae were found hiding than were found in the dark without plants.

To test the effect of plant volatiles directly, the researchers exposed larvae—some in the light and some in the dark—to a flow of volatiles collected from both uninfested and infested corn plants in light and dark conditions. When larvae in the dark were exposed to volatiles from uninfested plants, they hid in far greater numbers when the volatiles came from plants in the light than when they came from plants in the dark. And when larvae were in the light, far more hid when exposed to volatiles taken from plants in the light. Larvae responded similarly to volatiles taken from infested plants, though volatiles from infested plants in the light sent even more larvae into hiding.

These results demonstrate that it is not light that's controlling larval diurnal and nocturnal activity but volatiles released by the corn. Volatile compounds released during the day encourage hiding while those released at night indicate that it's safe to come out and eat. Just as parasitic wasps use plant volatiles to home in on potential victims, caterpillars use variations in their host plant's volatile production to reduce the risk of unpleasant encounters with wasps. Now that they've established volatiles' importance in influencing foraging behavior, the researchers plan to determine which compounds are responsible—and just how common insect–plant communication may be.

## 

**Figure:**
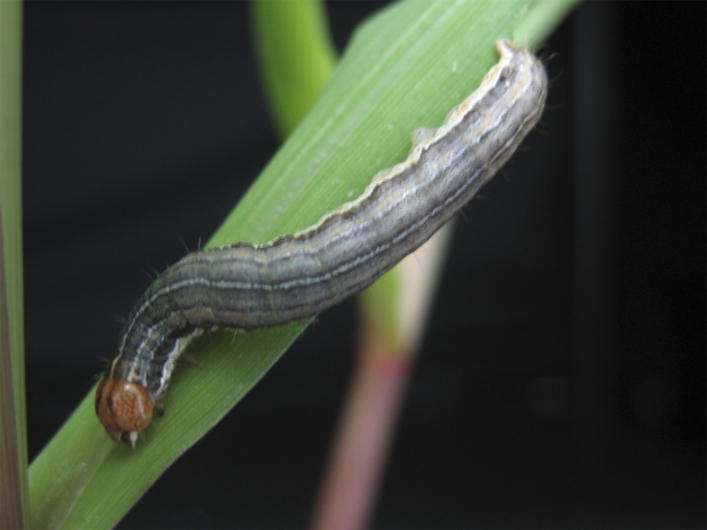
Plant volatiles released by corn plants may help the caterpillar M. separata avoid predators.

